# A Review of the Diversity of the Genital Tract Microbiome and Implications for Fertility of Cattle

**DOI:** 10.3390/ani12040460

**Published:** 2022-02-13

**Authors:** Mounir Adnane, Aspinas Chapwanya

**Affiliations:** 1 Institute of Veterinary Sciences, University of Tiaret, Tiaret 14000, Algeria; 2 Department of Clinical Sciences, Ross University School of Veterinary Medicine, West Indies, Basseterre 00265, Saint Kitts and Nevis; achapwanya@rossvet.edu.kn

**Keywords:** genital, microbiome, health, cattle, pathogens

## Abstract

**Simple Summary:**

Reproductive systems of cattle contain multiple microbes resident in the female from a young age. Sometimes other harmful microbes can invade the genital tract and cause diseases that impair fertility. Normally, commensal microbes facilitate genital tract homeostasis and produce factors that stimulate male sexual response. For this reason, the type and number of microbes present in the genital tract are important for reproductive tract health, and any disruption of this microbial balance leads to genital diseases. Interestingly, these microbes frequently populate the genital tract of cows, leading to reproductive diseases that perturb fertility. However, a microbiome composed of commensal microbes will likely result in the restoration of uterine health and improved fertility of the cows.

**Abstract:**

Cattle have a genital microbiome that is established early in life, even before calving. Microbial influx into the reproductive system of cows, during calving or mating, is unavoidable and is likely to alter the commensal microflora composition. It is now well established that a commensal endometrial flora is largely responsible for the overall fertility of cows. These microbes are important for maintenance of structural integrity of the genital mucosal barrier, immunomodulation, and protection against pathogens. Further, the genital microbiome functions in the semiochemical communication between a male and female. An optimal balance between the abundance and diversity of the microbiome is essential to promote female genital tract health. Disruption of this balance leads to dysbiosis and genital diseases and perturbed fertility. As part of the global strategy of One World, One Health, there is a need to reduce antibiotic use in animals. This area of research has the potential to expand the knowledge about the nexus between the endometrial microbiome and fertility including being probiotic in different species.

## 1. Introduction

The term microbiota refers to the entire population of microorganisms that colonizes a particular location and includes not just bacteria, but also other microbes such as fungi, archaea, viruses, and protozoans [1]. Cows have bacteria inhabiting the uterus even before calving and establish a unique endometrial microbiome within 20 min of calving where the microbiome is similar between cows that develop metritis and cows without endometritis until at least the second day postpartum. The microbiome of cows that develop metritis has higher relative abundance of *Bacteroidetes* and *Fusobacteria* and lower relative abundance of *Proteobacteria* and *Tenericutes* [2]. Furthermore, a mixture of bacteria, protozoa, fungi, and viruses is present in the genital tract of different species, including cattle [3]. While uterine infection is, in general, caused by mixed bacterial infections, the main microbial pathogens involved include *Trueperella pyogenes (T. pyogenes)*, *Fusobacterium necrophorum (F. necrophorum), Bacteroides* spp., and *Prevotella* spp. [4,5]. Microbial populations of the genital tract are highly variable, and *Lactobacillus* and *Bacteroides* are the most predominant in vaginal flora of women and cows, respectively [6,7]. The uterus has a unique microbiome, especially during pregnancy when the cervical contents are isolated from the vaginal contents as a result of the cervical mucus plug that is present during this period [8]. 

Significant interest has evolved for the genital tract microbiota in recent years within the scientific community such that the genital microbiota is thought to be associated with infertility or uterine diseases. The genital microbiome has important functions in the female reproductive tract through differential microbial competition. Symbiotic microorganisms create a biofilm that complements the cervicovaginal mucus, thereby protecting the genital tract from invasion by pathogens [9,10]. Furthermore, commensal microbes produce bioactive molecules such as lactic acid and reactive oxygen species that inhibit proliferation of pathogens [11]. During pregnancy, some *Lactobacilli* species confer protection of the offspring and are also associated with normal delivery [12]. Some recent studies focused on other aspects of reproductive function of cattle influenced by the genital microbiome such as pheromone production and semiochemical signaling [13]. 

**Figure 1:**
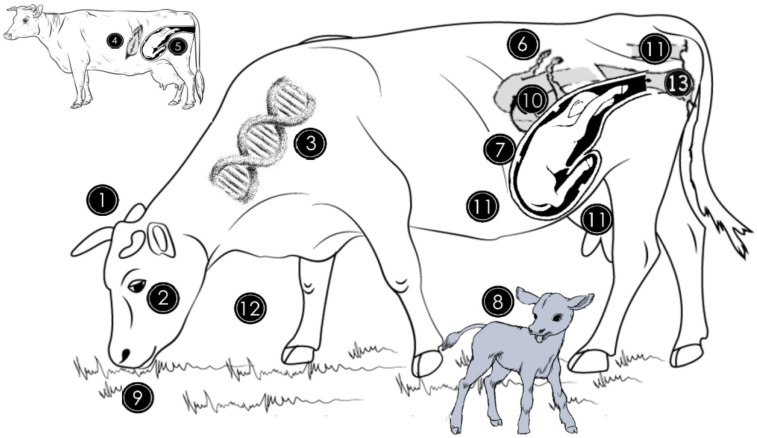
Origin of genital microbiome and factors that can affect the abundance and diversity of the microbial population. The genital microbiome is highly variable between species (1) and individuals of the same species (2). For instance, in cattle, genital microbiota is different between Gyr, Nellore, and Holstein breeds (3). The general microbiome of the newborn is similar to the mother’s skin microbiome if the delivery method was caesarean section (4) and is similar to the vaginal microbiome if it was natural delivery (5). Estrogen and progesterone hormone concentration variations during the estrous cycle influence bacterial growth in the genitalia by favoring some species at different times (6). During pregnancy, bacterial quantity and diversity decrease while archaeal abundance increases in vaginal milieu (7). Vaginal and uterine microbiomes of cows not diagnosed with metritis during the first month postpartum are similar in cows without uterine infections but differ from those with uterine infections (8). Dietary quality and quantity peripartum alter the endometrial microbiome through the provision of energy and protein nutrients (9). The uterine microbiome between 10 and 35 days postpartum is similar in cows not diagnosed with subclinical endometritis and those that will develop subclinical endometritis (10). Rumen, skin, rectum or feces (11) contribute to the establishment of the genital microbiome, while the environment (12) and intravaginal antibiotic therapy (13) can also alter the endometrial microbiota during a female’s lifetime.

While the genital microbes are thought to originate from the environment or different organs such as the rumen, skin, rectum or feces, the vagina is considered to be the main source of endometrial microfauna, especially during the times when the cervical lumen is less restricted at estrus, breeding or parturition (Figure 1) [8]. Vaginal mucus is less viscous during these time periods, allowing the uterus to be colonized by various bacteria, fungi, viruses, and protozoans of vaginal origin [3]. It is noteworthy that microbes can also access the genital tract hematogenously [14]. During calving, microbes prevalent in the environment influx into the uterus of cows. From an immunological perspective, these microorganisms are recognized as being pathogenic by the host immune defense system, thereby inducing a response to eliminate the pathogens. Typically, in cattle there is activation of the host defense system for removal the bacteria during endometrial involution within the first five weeks after calving [15]. It seems that the genital microbiome diversity is affected by many factors, some of, which are specific to the female such as estrous cyclicity [16] and pregnancy [17]. However, extrinsic factors such as nutrition [18] and genital pathologies [16,19] are equally important. The majority of the genital tract bacteria are non-pathogenic and are present with the enterocytes in a symbiotic relationship.

In humans, alteration of normal microbial diversity can lead to dysbiosis, infertility [20] or genital diseases such as vulvovaginal candidiasis [21]. There have been some studies conducted in animals on dysbiosis of the genital tract [17,22]. In this review paper, the focus was on the genital microbiome in cattle and how it has similarities to the human microbiome that has been extensively interrogated in relation to genital infections and infertility. While this paper is not a systematic review, there was a search and selection of all possible and recently published papers with relevant information and in direct relation to the topics of this paper. Because the microbiome has been investigated to a greater extent in humans, it was useful to include findings from some papers in this review with the most relevant results that can be applied in the animal field. In this review, we focus on the origin, diversity, and clinical relevance of microbiota and the risk for uterine disease or infertility of cattle. We also explore how this information can inform strategies such as pro- and prebiotics in combating uterine diseases.

## 2. The Genital Tract Contains a Dynamic Microbiota

The traditional dogma is that the endometrial environment is sterile, particularly during pregnancy [23]. It is now known that during calving, microbes present in calving areas can gain access to the uterus of the cow [24,25]. To identify the invading bacterial species, laboratories routinely utilize culture-based microbiological assays [26]. The advent and rapidly growing interest and availability of gene sequencing techniques mean that microbiome data using broad evaluation approaches are now readily available. Findings when using the independent culture methods of sequencing are revealing that the uterus of cattle contains a dynamic microflora [8,26,27]. Even during pregnancy, when the cervical plug is present, resulting in an isolation of the vaginal from the uterine microbiome, the uterus of cattle contains a unique microbiome during gestation [26]. The breakthrough findings that the human neonate is exposed to diverse bacterial species, originating from different sources depending on the type of delivery, paved the way for new discoveries in animals [28].

In cattle and sheep, *Bacteroidetes*, *Fusobacteria*, and *Proteobacteria* are the most common phyla in the genital tract [10]. At the genera level, *Aggregatibacter* spp., and *Streptobacillus* spp. were the most abundant. Interestingly, *Lactobacillus* spp., the predominate microbial populations in the human genital tract, were present in 80% and 90% of vaginal samples from ewes and cows, respectively [7,10,29]. In women, where the genital microbiome is intensively investigated, there are different vaginal microbial community state types (CSTs). The CSTs are defined by the dominant *Lactobacillus* species; CST I: *Lactobacillus crispatus* (*L. crispatus*), CST II: *L. gasseri*, CST III: *L. iners,* and CST V: *L. jensenni*. The CST IV population is defined by the absence of *Lactobacilli* species and the large diversity with strict and facultative anaerobic bacteria [7]. Such groupings, if present in cattle, may be indicative of bacterial profiles beneficial for fertility.

## 3. Significance of the Genital Microbiome

The reproductive tract microbiome of cattle is relatively under-explored, particularly in terms of the specific taxonomic classification and functional aspect of the microbiome, which are beneficial for the development of diagnostic methods, such as microbial biomarkers and dysbiosis indexes. The genital microbes in humans and animals have protective functions against major pathogens. In women, *Lactobacilli* produces lactic acid, which regulates vaginal pH and inhibits the proliferation of pathogens [30]. In turn, symbiotic bacteria utilize the secretions of the genital tract such as mucus sugar and proteins as a source of essential nutrients [11]. Lactic acid also induces acidification of milieu within the vagina, which interferes with intracellular functions, leading to microbial elimination [31]. Results from in vitro studies revealed that *Chlamydia trachomatis* is inactivated when there are normal concentrations of lactic acid [32], as are *Neisseria gonorrhoeae* and *Escherichia coli (E. coli)* [33,34]. One of the potential benefits of the commensal bacteria in women is the protection against human immunodeficiency virus type 1 (HIV-1). It seems that *Lactobacilli* in the vagina protect women from contracting HIV-1 during sexual intercourse. Lactic acid and the resultant biofilm limit the number of free virions and thereby reduce the shedding of HIV [35,36]. In vitro, HIV-1 is irreversibly inactivated when challenged with normal concentrations of lactic acid [37]. This may be the mechanism by which *Lactobacilli* confers vaginal protection against pathogens.

The genital microbiome is also implicated in the sociochemical signaling in different species through the production of pheromones [13,38,39]. Pheromones are produced by the microbiome by either direct production of pheromonal cues or through the fermentation hypothesis, by which microbes metabolize existing endogenous organic compounds to produce highly volatile compounds [40]. Results from a study in buffalo indicated there was a repetitive Flehmen response of males exposed to vaginal mucus from females at estrus [41]. Interestingly, the pheromones elaborated vary with the diversity of the genital microbiome [13,42]. 

## 4. Origin of the Genital Microbiome

The composition of the neonate microbiome is determined by the mode of birth and environment [28]. In humans, the general microbiome of the neonate delivered vaginally is similar to the maternal vaginal microbiome, dominated by *Lactobacillus*, *Prevotella*, or *Sneathia* spp. Neonates delivered through caesarean-section have a general microbiome similar to the maternal skin microbiome, mainly composed of *Staphylococcus*, *Corynebacterium,* and *Propionibacterium* spp. [28] (Figure 1). Therefore, initial exposure to the maternal microbiome influences an individual’s microbial load and diversity [28,43]. In cattle, microbes such *Bacteroides* and *Enterobacteriaceae* present in the genital tract causing reproductive diseases are thought to originate from the gut or the environment [44,45]. The results from some studies suggested that the vaginal microbiome originates from the gastrointestinal system [46], whereas others concluded that the vaginal microbial population includes methanogen species, and therefore the vaginal microbiome contributes to the establishment of the digestive tract microbiota [17]. The hematogenous route is also an important possible route for uterine contamination with pathogens [8].

## 5. Diversity of the Genital Microbiome

### 5.1. Vagina

Cows without uterine infections have in the vagina 15 taxa, predominantly *Bacteroides* (28.3%) and *Enterobacteriaceae* (17.8%), in addition to *Victivallis* (7.2%), *Streptococcus* (6.1%), *Phyromonadaceae* (5%), *Alistipes* (3.9%), *Coriobacteriaceae* (3.3%), *Clostridium* (3.3%), *Betaproteobacteria* (2.8%), *Corynebacterineae* (2.8%), *Cytophagaceae* (2.8%), *Oscillibacter* (2.8%), and *Planctomycetaceae* (2.8%) [6]. Cows with reproductive diseases such as purulent vaginal discharge have a more diverse vaginal microbiome containing 68 taxa, dominated by *Bacteroides* (35.83%), *Enterobacteriaceae* (18.62%), *Histophilus* (8.79%), *Alistipes* (4.34%), *Flavobacteriaceae* (1.77%), *Victivallis* (8.49%), *Coriobacteriaceae* (2.44%), *Streptococcus* (2.09%), *Barnesiella* (2.03%), and *Oscillibacter* (1.24%) (Table 1) [6]. Results from another study indicated that unclassified *Enterobacteriaceae* (21.05%), *Ureaplasma* (4.37%), and unclassified *Bacteroidaceae* (2.49%) were the most predominant [17]. At the phyla level, *Tenericutes* (36%), *Proteobacteria* (30%), *Fusobacteria* (7.6%), and *Firmicutes* (1.8%) were the most abundant [47]. In the study by Deng et al. (2019), *Firmicutes* (31.57%), *Proteobacteria* (24.08%), *Bacteroidetes* (12.96%), and *Tenericutes* (4.95%) were the most prevalent in the vagina. A comparison of the results from these two recent studies reveals that the proportions of the predominant microbial populations differ significantly between individuals.

### 5.2. Uterus

It is now known that cows have a natural microbiome in the uterus during the gestational period [26,59]. While the uterine microbes originate mainly from the vagina, and to a lesser extent, from the skin and gut, this microbiome is not as diverse as the vaginal microbiome [60]. Bacteria are always present in the uterus. *F. necrophorum*, *Porphyromonas Levii,* and *T. pyogenes* were detected in pregnant cows [26]. Interestingly, opportunistic microbes, such as *Histophilus* and *Mycoplasmataceae,* can become pathogenic [61,62]. Furthermore, the bacterial abundance in the uterus before calving is not associated with inflammation, which is indicative of a greater microbial tolerance during gestation [8].

## 6. Factors Affecting Genital Microbiome Diversity

The genital microbiome changes during the lifetime of females. Microbial populations in the reproductive tracts of animals are naturally selected because of different symbiotic functions. For example, in women, *Lactobacilli* use their small membrane extensions (i.e., fimbriae) that adhere to the genital mucosa [63]. Likewise, the vaginal tissue is rich in collagen, a valuable source of nutrients for *Aggregatibacter* spp. [9,64]. There are also other factors that affect the genital microbial diversity with some being specific to the stage of the female reproductive cycle, and others are extrinsic such as nutrition. Interestingly, the vaginal microbiome could have originated from the intestinal microbiota from an evolutionary perspective because there are marked similarities between the microbial population of the two anatomical parts [46]. The thought was that this microbial similarity is due to the fact that the vagina and anus are juxtaposed, and feces are often in contact with vulva [46]. However, our prevailing thoughts at present are that the genital microbiome of the neonate initially originated from maternal tissue that is in contact with the neonate subsequent to parturition, as described previously in this review article. The genital microbiome subsequently undergoes several changes during the lifetime of a female under the effect of the many factors, including the contamination by the microbiome of proximate organs such as the gastro-intestinal tract. Recent findings support our hypothesis (Figure 1) [17]. When there was a comparison of the changes in the microbial population in feces and vaginal samples collected before mating and at different stages of the gestational period, the fecal microbial diversity was the same, but the vaginal microbiome changed dynamically at different stages of the gestational period.

### 6.1. Intrinsic Factors

Individual variation in microbial species in the genital tract of bovids is thought to have effects of fertility outcomes [46]. This variation may explain how some animals develop resistance and others become infected with uterine diseases. Such differences are thought to be common in other mammals including humans (Figure 1) [1].

#### 6.1.1. Species

The genital microbiome is diverse among animal species and also individual animals, which perturbs the regulation of reproductive hormones (Figure 1) [10]. For example, in two separate studies using either cows with endometritis or those administrated bacterial lipopolysaccharide (LPS), estradiol concentrations were lower, and there was a relatively prolonged period to the time of ovulation during the follicular phase of the reproductive cycle [65,66]. The vaginal microbiomes of cattle were composed of a large abundance of *Enterococcus* spp., *Staphylococcus* spp., and *Streptococcus* spp., which was different from the vaginal microbiome of ewes where there was a predominance of *Bacillus* spp., *Corynebacterium* spp., *Escherichia* spp., *Staphylococcus* spp., and *Streptococcus* spp. (Table 1) [50,67,68,69]. At the phyla level, the genital microbiomes in both cows and ewes were composed predominantly of *Proteobacteria*, *Fusobacteria*, and *Bacteroidetes*. At the genera level, *Aggregatibacter* spp. and *Streptobacillus* spp. were the predominant species [10]. While *Lactobacilli* are detected in 80% of ewes and 90% of cows, the total microbial population is less abundant, leading to the near-neutral vaginal environment compared to the acid environment in women where there is a large population of *Lactobacilli* [10]. In addition to *Lactobacilli*, cows and ewes share several genera, mainly *Sneathia* spp., *Porphyromonas* spp., and *Prevotella* spp. [7,10]. A small *Firmicutes* to *Bacteroidetes* ratio is an early indicator of cows that subsequently develop postpartum endometritis [54].

#### 6.1.2. Breed (Genetic Background)

In Gyr cattle, a common dairy breed in South American countries such as Brazil, the vaginal microbiome is enriched with bacteria and fungi while there is a small population of archaea (Figure 1) [49]. Among bacteria, *Firmicutes*, *Bacteroidetes*, *Proteobacteria*, and *Actinobacteria* were the most frequently detected. *Mycosphaerella* and *Cladosporium* were the most frequently detected fungal genera. While archaea were in low abundance, the *Methanobrevibacter* genus was the most abundant (Table 1). In Nellore beef cattle, the vaginal microbiome is predominately composed of *Firmicutes*, *Bacteroidetes*, *Proteobacteria,* and up to 20% of unclassified bacteria [46]. *Mycosphaerella* was the most abundant fungal genus while *Methanobrevibacter* was the predominant archaeal genus. In Holstein Friesian cattle, the most ubiquitous dairy breed in North Africa, Europe, and the USA, the vaginal microbiome was predominately composed of *Firmicutes*, *Tenericutes*, *Proteobacteria*, and *Bacteriodetes* phyla. Other bacteria were detected in smaller quantities such as *Actinobacteria* and *Spirochaetae* [48].

#### 6.1.3. Delivery Mode

The type of fetal delivery at parturition is one of the major factors affecting the genital microbiome diversity [28]. The general microbiome of the neonate is similar to the maternal skin microbiome if the parturition was by cesarean section and similar to the vaginal microbiome if it was natural delivery (Figure 1) [28]. Early established bacterial communities provide protection against pathogenic bacteria that may be infective agents to the neonate. While the maternal vagina is the main source for the natural microbiome, a unique neonatal microbiome is established shortly after parturition with microbes from other sources. For example, neonates delivered by caesarean section have a greater prevalence of methicillin-resistant *Staphylococcus aureus (S. aureus)* (MRSA) skin infections (64–82%) compared to when neonates are born without complications (i.e., natural delivery) [28].

#### 6.1.4. Estrous Cyclicity 

Estrous cycles in cattle are regulated by hormonal concentration changes with relatively higher concentrations of estradiol during proestrus and estrus and relatively higher progesterone concentrations during metestrus and diestrus, having marked effects on the vaginal pH in mammals [70,71]. Microbes are very sensitive to acidic milieu; therefore, it is thought that when there are greater estradiol concentrations, there will be effects on the microbiome in the genital milieu of some species, with effects varying as a result of estradiol concentrations (Figure 1). Results from a recent study indicated that *Bacteroidete* spp., *Histophilus somni, Actinobacillus seminist,* and unclassified *Fusobacterium* populations increase when there are relatively lower estradiol concentrations in the vaginal milieu (Table 1) [47]. When there are relatively higher estradiol concentrations in the vaginal milieu, unclassified *Pasteurellaceae* are the predominant microbes in the vagina. Likewise, when there are higher progesterone concentrations, the populations of microbes in the vagina are relatively larger [46,50]. 

#### 6.1.5. Pregnancy 

During the gestational period, there is lower microbial diversity of the vaginal microbiome and a greater archaeal population [46]. The bacterial species present in the vagina during the gestational period are less diverse due to the relatively higher progesterone concentrations that lead to suppression of the vaginal microbial population (Figure 1) [50]. The relatively lower microbial population in the vagina during the gestational period could lead to a greater risk of dysbiosis and abortion. Similarly, in humans, the microbial population in the vagina decreases as duration of the gestational period increases [72,73]. Unlike in humans, the vaginal microbiome of cattle is relatively stable throughout the gestational period [17]. In nonpregnant heifers, *Pasteurellaceae* spp. and *Fusobacterium* spp. were abundant in the vagina, whereas pregnant heifers had a greater prevalence of *Pasteurella multocida* (Table 1) [47]. 

#### 6.1.6. Postpartum

A large variation among individuals was reported in the uterine microbiome of cows without uterine infections during the first month postpartum [53,54]. Alpha and beta diversities were not affected by the postpartum day of sampling with the bacterial diversity being similar among 10, 21, and 35 days postpartum (DPP) in the uterus of cows without uterine inflammation and those with uterine inflammation; however, the uterine microbiome was markedly different between the two groups (Figure 1) [53]. Based on results from a metagenomic analysis of uterine simples collected three times during the first 35 DPP, the uterine microbiome of cows without uterine inflammation was predominantly composed of *Porphyromonas*, *Bacillus*, *Schlegelella*, *Paracoccus,* and *Fusobacterium* (Table 1) [53]. 

Interestingly, the vaginal, and uterine microbiomes of cows without uterine inflammation during the first 50 DPP were similar and were highly enriched with *Firmicutes* [54]. This finding could be explained by the fact that soon after calving, the cervical lumen is less constricted, resulting in a mixing of the vaginal and uterine milieu with there being movement of these contents throughout the reproductive tract. We hypothesize that in cows without uterine inflammation, the genital microbiome is not contaminated during calving by the external microbiome or at least not affected for an extended period subsequent to parturition. A comparison of the genital microbiome of pregnant cows before calving retrospectively with those that develop endometritis after 21 DPP to those that continue to have an uninfected uterus revealed that genital microbiome pre-calving was similar to the vaginal microbiome of healthy cows that did not develop uterine infections subsequent to 21 DPP [54]. 

### 6.2. Extrinsic Factors

#### 6.2.1. Nutrition 

The microbial population of the uterus of dairy cows during the postpartum period was reported to be affected by the nutrient content of the diet, especially the energy content, around the time of calving (Figure 1) [18]. Cows that were fed 80% of the energy requirement had uterine inflammation, and the predominant species of the uterine microbiome were *Bacteroidetes* and *Fusobacteria* (Table 1). A comparison of the uterine microbiome pre-calving to that post-calving in cows that developed metritis with those that did not contract metritis indicated that cows with metritis had predominantly *Bacteroides* and *Fusobacteria* and a lower abundance of *Proteobacteria* and *Tenericutes*, which was markedly different from those that did not have metritis [8]. Therefore, nutrition affects the genital microbiome through the modulation of general metabolism and immune functions and therefore affects the occurrence of dysbiosis and genital infections.

#### 6.2.2. Genital Pathologies

Interestingly, cows with subclinical endometritis had a similar uterine microbiome compared to cows without uterine inflammation between 10 and 35 DPP; however, cows with clinical endometritis had a microbiome with a different composition compared to the two other groups (Figure 1) [53]. Cows affected with clinical endometritis had a lower bacterial diversity characterized by a greater prevalence of *Fusobacterium* and *Trueperella* associated with a lower abundance of *Escherichia, Shigella, Lactobacillus, Prevotella, Schlegelella*, and *Streptococcus* compared to cows without uterine inflammation and those with subclinical endometritis (Table 1) [53,54]. This latter group had relatively a greater prevalence of *Anaerococcus*, *Corynebacterium*, and *Staphylococcus* compared to cows with clinical endometritis.

The microbial population of the uterus and the vagina at 7 DPP is associated with the risk of developing clinical endometritis after 21 DPP [54]. The vaginal microbiome at 7 DPP in cows with endometritis is highly enriched with *Bacteroides*, *Helcococcus,* and *Fusobacterium*, unlike cows without uterine inflammation where *Firmicutes* are the most predominant. Comparisons of the uterine and vaginal microbiomes in these animals indicated that at 7 DPP, the microbial composition was similar between the two compartments, but this similarity was less between 21 and 50 DPP [54]. These findings confirm that the genital microbiome of cows at risk of developing endometritis is not completely established during first month postpartum, unlike in cows without uterine inflammation where the uterine and vaginal microbiomes are well established at day 7 postpartum [54].

Metritis, which is indicated by a marked inflammation of the endometrium and myometrium soon after calving and before day 21 postpartum, is characterized by relatively lower abundance of the vaginal microbiome quantities and higher relative abundance of *Bacteroides*, *Porphyromonas*, and *Fusobacterium* (Table 1) [8,55]. Likewise, cows with metritis had a uterine microbiome that was markedly enriched with *F. necrophorum*, *Porphyromonas levii*, and *Prevotella melaninogenica* compared with cows without metritis [56]. *E. coli* is another interesting bacterium that is detected early postpartum in most of the cows because the presence of this microbe is important for the development of *F. necrophorum,* leading to metritis, while the latter bacterium is often detected in association with *T. pyogenes* in the case of endometritis [8,57]. In women with mild or moderate vaginal atrophy, the vaginal microbial population tends to be predominantly invasive pathogens, mainly *Streptococcus* and *Prevotella* [74]. Microbiomes in cattle diagnosed with necrotic vulvovaginitis (BNVV), as compared with those not diagnosed with BNVV, are characterized by a relatively microbial diversity and a large abundance of *Bacateroidetes* [58].

## 7. Dysbiosis and Genital Microbiome Disruption

Bacterial dysbiosis is an inflammatory condition characterized by the presence of polymicrobial populations of commensal microbes in moderate abundance or the complete absence of these commensal microbes. In humans, bacterial vaginosis, a type of dysbiosis, is characterized by the predominance of specific species of strict and facultative anaerobic microbes associated with a lower abundance or complete absence of *Lactobacilli* and larger microbial population in the vaginal microbiome [72,75]. Results from a study conducted to investigate whether microbes that were predominant in the vagina before mating could be predictive of females that will become pregnant revealed that *Histophilus somni*, *Clostridiaceae* 02d06, and *Campylobacter* were abundant in cows that failed to conceive (Table 1) [17]. Commensal bacteria present in the genital tract, mainly the vagina in females without uterine inflammation, have important functions in the genital tract by suppressing infection by major pathogens by different mechanisms, mainly through the competition effect by the occupation of specific receptors to which pathogenic microbes bind to gain entry into cells and by secreting immune active molecules such as lactic acid [11]. Interestingly, the absence or decreased abundance of *Lactobacilli* in the genital tract of pregnant women was associated with a lower vaginal pH, leading to overgrowth of more pathogenic microbes [7,76]. The inflammatory reaction, therefore, is induced, and interleukin 8 (IL-8) is secreted in large quantities, leading to abortion or premature parturition [76]. In pregnant cows, the size of the population of *S. aureus* was associated with the risk of abortion, while this pathogen was rarely isolated from cows that had a typical length of gestation [51]. *T. pyogenes* is another major pathogen associated with abortion during the last half of gestation [52]. While *Fusobacteria* and *Bacteroidetes* are common flora of the genital tract in cows with or without genital tract inflammation, preventing the overgrowth of these bacteria is important for preventing reproductive tract infections [8]. Intravaginal antibiotic treatment affects the genital microbiome, leading to dysbiosis. Bitches administrated antibiotics intravaginally during estrus were less likely to attract males when in estrus compared with females not treated with antibiotics (Figure 1) [77]. The changes in the sexual behavior would be the result of changes in the microbial diversity and the semiochemical signal emitted by the modified microbiome because of the antibiotic treatment.

## 8. Genital Microbial Population as Probiotic Treatment for Dysbiosis

Treating reproductive diseases in cattle is a challenge for veterinarians and farmers. For example, antibiotic treatment of metritis results in only 67 to 77% of the cases recovering from clinical symptoms, yet fertility continues to be compromised [78]. Modulating the genital microbiome using probiotics is becoming an effective strategy in humans [21,79,80]. The administration of lactic acid bacteria intravaginally resulted in modification of the uterine microbiome [81] while intravaginal administration of *L. plantarum* P17630 prevented recurrent vulvovaginal candidiasis [21]. Likewise, *L. crispatus* administrated intravaginally was effective in the management of recurrent urinary tract infections [79].

In cattle, intravaginal treatment with a mixture of lactic acid bacteria, *L. rhamnosus* CECT 278, *Pediococcus acidilactici* CECT 5,915, and *L. reuteri* DSM 20016, three weeks before calving resulted in decreased metritis prevalence by 58% [80]. The probiotic treatment using lactic acid bacteria modulates the inflammatory reaction by downregulating the relative abundance of the *L-selectin* mRNA transcript, which encodes for this protein. The L-selectin protein has been implicated in the neutrophil infiltration into the infected tissue and expression of the tumor necrosis factor receptor (*TNF-R*) gene that modulates degranulation and phagocytic processes [80,82,83]. This reduction in neutrophil activity could be the result of reduced pathogenic bacteria in the genital tract due to a competitive effect of lactic acid bacteria or by coaggregation with more pathogenic microbes reducing the adhesion of these pathogens to specific receptors on the cell surface [80,84]. Results from in vivo and ex vitro studies revealed that the association of *L. rhamnosus*, *Pediococcus acidilactici*, and *L. reuteri* resulted in a marked reduction of the inflammatory profile of endometrial epithelial cells when *E. coli* was administered or included in the culture medium [85]. Likewise, the growth of *S. aureus*, one of the major pathogens implicated in postpartum infections in dairy cattle, was inhibited in vitro under treatment with *L. gasseri* CRL1421 and CRL1412 [86].

## 9. Conclusions

The genital microbiota represents an opportunistic field of study in the realm of cattle fertility when considering the microbiome is an essential immunological barrier against pathogens and for pheromone production. Specific questions that can be addressed are the functions of specific bacteria taxa involved in physiological uterine involution in postpartum cows. With dysbiosis, uterine disease can occur; thus, modulating the vaginal microbiome may be an effective alternative to antibiotic therapies. Therefore, intravaginal inoculation of cattle at risk of infection represents possible probiotic management of genital postpartum uterine infections.

## Figures and Tables

**Table 1:** Richness and diversity of the vaginal microbiome in cattle.

Factors		Genital Microbiome Composition	References
Species	*Bos taurus*	Phylum: *Proteobacteria*, *Fusobacteria*, and *Bacteroidetes*. Genera: *Aggregatibacter* spp., and *Streptobacillus* spp. *Lactobacilli*, *Sneathia* spp., *Porphyromonas* spp., and *Prevotella* spp.	[7,10]
Breed	Holstein	Phylum: *Firmicutes*, *Tenericutes*, *Proteobacteria*, and *Bacteriodetes Actinobacteria* and *Spirochaetae*	[48]
	Gyr	Bacteria genus: *Firmicutes*, *Bacteroidetes*, *Proteobacteria*, and *Actinobacteria* Fungal genus: *Mycosphaerella* and *Cladosporium*. Archaea genus: *Methanobrevibacter* genus.	[49]
	Nellore	*Bacteria: Firmicutes*, *Bacteroidetes*, *Proteobacteria,* and up to 20% of the unclassified bacteria fungal genus: *Mycosphaerella* and Archaeal genus: *Methanobrevibacter*	[46]
Estrous cyclicity	Follicular phase (high estradiol)	*Pasteurellaceae unclassified*	[47]
	Luteal phase (Low estradiol)	Larger microbial diversity	[46,47,50]
		*Bacteroidete* spp., *Histophilus somni, Actinobacillus seminis,* and unclassified *Fusobacterium*	[47]
Pregnancy	Pregnant	Microbiome is relatively stable throughout the gestational period	[17]
		*Pasteurella multocida*	[47]
	Non pregnant	*Pasteurellaceae* spp. *Fusobacterium* spp.	[47]
		*Histophilus somni, Clostridiaceae 02d06,* and *Campylobacter*	[17]
	Abortion	*Staphylococcus aureus*	[51]
		*Trueperella pyogenes*	[52]
Postpartum	First 35 days postpartum	*Porphyromonas, Bacillus, Schlegelella, Paracoccus,* and *Fusobacterium*	[53]
	First 50 days postpartum	Similar between individuals Enriched with *Firmicutes*	[54]
Uterine disease	Without uterine disease	*Bacteroides* (28.3%), *Enterobacteriaceae* (17.8%), *Victivallis* (7.2%), *Streptococcus* (6.1%), *Phyromonadaceae* (5%), *Alistipes* (3.9%), *Coriobacteriaceae* (3.3%), *Clostridium* (3.3%), *Betaproteobacteria* (2.8%), *Corynebacterineae* (2.8%), *Cytophagaceae* (2.8%), *Oscillibacter* (2.8%), and *Planctomycetaceae* (2.8%)	[6]
		Unclassified *Enterobacteriaceae* (21.05%), *Ureaplasma* (4.37%), and unclassified *Bacteroidaceae* (2.49%) *Firmicutes* (31.57%), *Proteobacteria* (24.08%), *Bacteroidetes* (12.96%), and *Tenericutes* (4.95%)	[17]
		Phylum: *Tenericutes* (36%), *Proteobacteria* (30%), *Fusobacteria* (7.6%), and *Firmicutes* (1.8%)	[47].
		*Firmicutes* are the most predominant	[54].
	With uterine diseases (all included)	*Bacteroides* (35.83%), *Enterobacteriaceae* (18.62%), *Histophilus* (8.79%), *Alistipes* (4.34%), *Flavobacteriaceae* (1.77%), *Victivallis* (8.49%), *Coriobacteriaceae* (2.44%), *Streptococcus* (2.09%), *Barnesiella* (2.03%), and *Oscillibacter* (1.24%)	[6]
		Small *Firmicutes* to *Bacteroidetes* ratio	[54]
		Enriched with *Bacteroides*, *Helcococcus,* and *Fusobacterium*	
	Metritis	Lower microbial diversity Higher relative abundance of *Bacteroides*, *Porphyromonas*, and *Fusobacterium*	[8,55]
		Enriched with *Fusobacterium necrophorum*, *Porphyromonas levii*, and *Prevotella melaninogenica*	[56]
		Enriched with *Escherichia coli*	[8,57]
	Clinical endometritis	Lower bacterial diversity High prevalence of *Fusobacterium* and *Trueperella* Lower abundance of *Escherichia, Shigella, Lactobacillus, Prevotella, Schlegelella*, and *Streptococcus*	[53,54]
	Subclinical endometritis	Greater prevalence of *Anaerococcus*, *Corynebacterium*, and *Staphylococcus*	[53,54]
	Necrotic vulvovaginitis	Decreased microbial diversity Large abundance of *Bacateroidetes*	[58]
Nutrition	Energy deficiency around calving	High prevalence of *Bacteroidetes* and *Fusobacteria*	[18]

## Data Availability

Not applicable.
